# Low-Frequency Harmonic Perturbations Drive Protein Conformational Changes

**DOI:** 10.3390/ijms221910501

**Published:** 2021-09-28

**Authors:** Domenico Scaramozzino, Gianfranco Piana, Giuseppe Lacidogna, Alberto Carpinteri

**Affiliations:** 1 Department of Structural, Geotechnical and Building Engineering, Politecnico di Torino, Corso Duca degli Abruzzi 24, 10129 Torino, Italy; gianfranco.piana@polito.it (G.P.); giuseppe.lacidogna@polito.it (G.L.); alberto.carpinteri@polito.it (A.C.); 2 Department of Bridge Engineering, Tongji University, 1239 Siping Road, Shanghai 200092, China; 3 Department of Civil and Environmental Engineering, Shantou University, Shantou 515063, China

**Keywords:** protein dynamics, low-frequency vibrations, modal analysis, elastic network model, harmonic perturbation, conformational change, principal component analysis

## Abstract

Protein dynamics has been investigated since almost half a century, as it is believed to constitute the fundamental connection between structure and function. Elastic network models (ENMs) have been widely used to predict protein dynamics, flexibility and the biological mechanism, from which remarkable results have been found regarding the prediction of protein conformational changes. Starting from the knowledge of the reference structure only, these conformational changes have been usually predicted either by looking at the individual mode shapes of vibrations (i.e., by considering the free vibrations of the ENM) or by applying static perturbations to the protein network (i.e., by considering a linear response theory). In this paper, we put together the two previous approaches and evaluate the complete protein response under the application of dynamic perturbations. Harmonic forces with random directions are applied to the protein ENM, which are meant to simulate the single frequency-dependent components of the collisions of the surrounding particles, and the protein response is computed by solving the dynamic equations in the underdamped regime, where mass, viscous damping and elastic stiffness contributions are explicitly taken into account. The obtained motion is investigated both in the coordinate space and in the sub-space of principal components (PCs). The results show that the application of perturbations in the low-frequency range is able to drive the protein conformational change, leading to remarkably high values of direction similarity. Eventually, this suggests that protein conformational change might be triggered by external collisions and favored by the inherent low-frequency dynamics of the protein structure.

## 1. Introduction

Proteins affect virtually every biological process occurring in the human body [1]. Their correct functioning is pivotal for a variety of tasks, such as delivery of nutrients throughout and across cells, recognition and neutralization of pathogenic bacteria and viruses, providing of suitable strength and rigidity to tissues, activation of signaling pathways and catalytic reactions, etc. [2]. All these activities are performed within the physiological environment and in a highly dynamic fashion. This explains why so much research has been carried out in the last decades regarding protein dynamics and its relationship with the biological functionality. One of the main computational approaches used to investigate protein dynamics is molecular dynamics (MD) [3,4]. MD is based on the numerical integration of Newton’s laws of motion of the molecular system under scrutiny, subjected to the forces arising from the gradients of the interatomic potentials [5]. Despite the high potential of MD simulations, its applicability to large peptide chains and protein complexes, especially for the investigation of the large-scale slow dynamics, remains quite elusive and requires cautious analysis of the results.

While trying to overcome the limitations of MD simulations and come up with more simplified approaches which could be of value for a general understanding of protein functionality, it was found that elastic models based on single-parameter Hookean potentials are still able to describe the slow protein dynamics in good detail [6]. These models treat the protein as a network of elastic springs, connecting the atoms whose positions in the reference structure are assumed to be at the equilibrium, around which the thermal fluctuations take place [7,8,9]. Despite the simplicity of this model, the predicted fluctuations as well as the obtained vibrational frequencies were found in good agreement with those obtained by considering more complex semi-empirical potentials [6]. This discovery paved the way for the development of the coarse-grained elastic network models (ENMs), such as the Gaussian network model (GNM) [10,11,12,13,14,15,16] and the anisotropic network model (ANM) [17]. The GNM assumes that the protein structure undergoes isotropic fluctuations around the equilibrium position, therefore it predicts the amplitude of these fluctuations and hence it can be identified as a unidimensional model. Conversely, the ANM takes also into consideration the directionality of the expected motion, the protein structure being modelled as an actual three-dimensional network.

The ANM was extensively used for the investigation of protein dynamics for three main reasons. Firstly, the computed fluctuations are found to exhibit a good agreement with the B-factors obtained from crystallographic experiments, thus providing good estimates of the protein flexibility [17,18,19,20,21,22,23]. Secondly, the ANM low-frequency motions are found to describe fairly accurately the directionality of the protein conformational change [20,24,25,26,27,28,29,30,31,32,33,34]. These conformational changes usually occur when the protein switches its three-dimensional shape while performing its biological activity (e.g., during ligand-binding or phosphorylation phenomena) and, therefore, they are informative of the protein biological mechanism [1]. Thirdly, this model allows to obtain useful insights on the low-frequency dynamics with small computational burden, especially if compared with the more time-consuming MD simulations.

From a structural mechanics viewpoint, we have recently shown that the ANM can be seen as a spatial truss elastic model, where the atoms of the protein network can be replaced by frictionless spherical hinges and the Hookean connections by linear elastic bars [35,36]. In the traditional formulation of the ANM, the Hessian matrix of the network is computed and diagonalized to obtain the eigenvalues and eigenvectors. The former are associated with the vibrational frequencies, while the latter identify the mode shapes of vibration [17]. However, since the mass of the protein is not explicitly taken into account in the classical ANM, the eigenvalues are only qualitatively related to the vibrational frequencies. In our previous works, we have also added the explicit mass information, thus obtaining more quantitative information about the frequencies of vibration via a classical free-vibration modal analysis [35,36]. In particular, in the case of lysozyme, we observed that the lowest-frequency modes lie in the sub-THz frequency range, with frequency values of the order of few tens of GHz, in agreement with previous studies [6,37,38,39,40].

Another powerful application of the ANM, which has been developed since the last decade, is based on application of perturbations on the protein elastic network, both to probe protein flexibility and conformational changes. Eyal and Bahar [41] developed a methodology that made use of the ANM normal modes to assess the anisotropic mechanical resistance of proteins under external pulling forces. This analysis was able to explain the anisotropy of the mechanical resistance observed from single-molecule manipulation techniques, such as atomic force microscopy (AFM). More recently, we made use of two structural metrics, which are well-known in the field of structural mechanics (i.e., compliance and stiffness), to study the flexibility of protein structures under pairwise force application [42]. These metrics enabled to predict the distribution of protein flexibility and rigidity throughout the protein chain and were verified against the experimental B-factors. Referring to protein conformational changes, Ikeguchi et al. [43] observed that protein transitions can be numerically simulated by evaluating the linear response of the protein reference structure subjected to external forces applied at specific locations. Based on this finding, the Atilgan’s group developed the perturbation-response scanning (PRS) technique, where directed forces are applied to the protein structure at single residues and the protein response is calculated and compared with the conformational change observed experimentally [44]. The method was shown to work well for the prediction of a variety of protein conformational changes [45], as well as for the detection of allosteric sites [46]. More recently, the PRS method was used by Liu et al. [47], coupled with an energy-based Metropolis Monte Carlo (MMC) algorithm, in order to simulate the complete closed-to-open transition of the GroEL subunit, induced by directional forces applied at the ATP-binding site.

From what we have reported above, it is evident that, when starting from the knowledge of the reference structure only, protein conformational changes have been analyzed with the ANM by following two separate approaches: (1) Evaluating the normal modes of vibration of the reference elastic model, with subsequent comparison between each individual mode shape and the conformational change; (2) applying forces to the protein reference structure and evaluating the response of the network in terms of displacements, with subsequent comparison with the observed conformational change. Fundamentally, approach (1) considers the free-vibration dynamics of the protein, whereas approach (2) focuses on the static response of the protein structure under external forces. In this work, we put the two approaches together, thus applying forces to the protein structure in a dynamic fashion. In this way, we exploit the main ideas behind both approaches: (1) Conformational changes might be favored by the intrinsic protein dynamics along its low-frequency modes of vibration [24,29]; (2) conformational changes might be triggered by external perturbations [43,45].

In particular, we apply external harmonic perturbations, randomly distributed in the space-domain but with a well-defined frequency content in the time-domain, to the protein ANM. The equations of motions are numerically solved, by considering mass, viscous damping and elastic stiffness contributions, in order to assess the complete time-dependent dynamic response of the protein. The time-history of nodal displacements is then evaluated both in the coordinate space, as well as in the sub-space of principal components (PCs) via the application of principal component analysis (PCA). The obtained time-dependent displacements are then compared to the observed conformational change, in order to find in which conditions these external perturbations are able to drive the conformational change. Results are shown here for lysine-arginine-ornithine(LAO)-binding protein, considering different perturbation patterns, damping coefficients and frequencies of excitation. The results of the analysis reveal that, when the external perturbation is applied in the low-frequency range, the protein structure undergoes a displacement field closely aligned with the observed conformational change, with a remarkably high overlap score (up to 0.95).

## 2. Methodology

In this section we describe the fundamentals of the ANM, starting from the calculation of the natural modes of vibration (Section 2.1), the evaluation of the protein response under external perturbations applied in a static fashion (Section 2.2) and, finally, how the two approaches can be put together in order to retrieve the complete protein response under time-dependent external perturbations (Section 2.3).

### 2.1. ANM Fundamentals and Free-Vibration Analysis

The anisotropic network model (ANM) treats the protein structure as a network of atoms connected by Hookean connections, which are meant to simulate the interatomic interactions in a simplified manner. In its traditional coarse-grained representation, C_α_ atoms are usually taken as the reference nodes for each amino acid [17]. The positions of these nodes can be taken from the coordinates of the crystal structure, which is available from the Protein Data Bank [48] and assumed to be the equilibrium state of the protein. Each pair of nodes *i* and *j* lying at a distance *r_i,j_* lower than a certain cutoff threshold *r_c_* are connected by a linear elastic spring, having a spring constant equal to *γ_i,j_*. Commonly employed values of *r_c_* are around 12–15 Å [17]. For each connection among nodes *i* and *j*, the Hessian (stiffness) matrix [*H_i,j_*] can be computed based on the values of the spring constant *γ_i,j_* and coordinates of atoms *i* and *j* [17,35]:

(1) $$\left[H_{i,j}\right]=-\frac{\gamma_{i,j}}{r_{i,j}{}^{2}}\left[\begin{array}{ccc} {\left(x_{j}-x_{i}\right)}^{2} & \left(x_{j}-x_{i}\right)\left(y_{j}-y_{i}\right) & \left(x_{j}-x_{i}\right)\left(z_{j}-z_{i}\right)\\ \left(x_{j}-x_{i}\right)\left(y_{j}-y_{i}\right) & {\left(y_{j}-y_{i}\right)}^{2} & \left(y_{j}-y_{i}\right)\left(z_{j}-z_{i}\right)\\ \left(x_{j}-x_{i}\right)\left(z_{j}-z_{i}\right) & \left(y_{j}-y_{i}\right)\left(z_{j}-z_{i}\right) & {\left(z_{j}-z_{i}\right)}^{2} \end{array}\right].$$

For a protein network made up of *N* nodes, the complete 3*N* × 3*N* Hessian matrix [*H*] can be computed as a Boolean combination of the sub-matrices [*H_i,j_*], i.e.,

(2) $$\left[H\right]=\left[\begin{array}{cccccc} \left[H_{1,1}\right] & \left[H_{1,2}\right] & \ldots & \left[H_{1,i}\right] & \ldots & \left[H_{1,N}\right]\\ \left[H_{2,1}\right] & \left[H_{2,2}\right] & \ldots & \left[H_{2,i}\right] & \ldots & \left[H_{2,N}\right]\\ \ldots & \ldots & \ldots & \ldots & \ldots & \ldots \\ \left[H_{i,1}\right] & \left[H_{i,2}\right] & \ldots & \left[H_{i,i}\right] & \ldots & \left[H_{i,N}\right]\\ \ldots & \ldots & \ldots & \ldots & \ldots & \ldots \\ \left[H_{N,1}\right] & \left[H_{N,2}\right] & \ldots & \left[H_{N,i}\right] & \ldots & \left[H_{N,N}\right] \end{array}\right],$$

where:

(3) $$\left[H_{i,i}\right]=-\overset{N}{\underset{j=1,j\neq i}{\sum }}\left[H_{i,j}\right].$$

At this point, the ANM looks for the eigenvalues and eigenvectors of [*H*]. The former are qualitatively associated with the fundamental frequencies of vibration, while the latter reflect the natural mode shapes of the protein network [17]. Notice that, since the protein is not externally constrained, the first six mode shapes are associated with rigid-body motions (translations + rotations) of the entire molecule at zero-frequency. Thus, the mode shapes accounting for the internal deformability of the protein are related to the subsequent 3*N* − 6 eigenvectors. These vibrational modes have been extensively used to analyze and predict protein conformational changes, and good agreement has often been found, especially for the most collective conformational transitions [24,27,28,29,30].

In order to have more quantitative information about the frequencies of vibration, the mass of the structure needs to be included into the calculations [35,36,49,50,51]. This can be easily done by considering the 3*N* × 3*N* mass matrix [*M*] of the network:

(4) $$\left[M\right]=\left[\begin{array}{cccccc} \left[M_{1,1}\right] & \left[0\right] & \ldots & \left[0\right] & \ldots & \left[0\right]\\ \left[0\right] & \left[M_{2,2}\right] & \ldots & \left[0\right] & \ldots & \left[0\right]\\ \ldots & \ldots & \ldots & \ldots & \ldots & \ldots \\ \left[0\right] & \left[0\right] & \ldots & \left[M_{i,i}\right] & \ldots & \left[0\right]\\ \ldots & \ldots & \ldots & \ldots & \ldots & \ldots \\ \left[0\right] & \left[0\right] & \ldots & \left[0\right] & \ldots & \left[M_{N,N}\right] \end{array}\right],$$

where [*M_i,i_*] = *m_i_*[*I*], *m_i_* being the mass of node *i* (e.g., equal to the actual mass of the *i*^th^ amino acid for a C_α_-only coarse-grained model), and [*I*] represents a 3 × 3 unitary matrix. The Hessian and mass matrices reported above can then be diagonalized together, following the well-known approach of modal analysis to retrieve the fundamental modes of vibration. This yields the fundamental eigenvalue-eigenvector equation [52]:

(5) $$\left(\left[H\right]-\omega_{n}{}^{2}\left[M\right]\right)\{\delta_{n}\}=\left\{0\right\},$$

where *ω_n_* is the angular frequency of vibration associated with mode *n*, and {*δ_n_*} the 3*N* × 1 vector containing the mass-weighted displacements associated to the *n*^th^ mode shape. Notice that *ω_n_* is related to the vibrational frequency *f_n_* by *f_n_* = *ω_n_*/2π. As in the traditional ANM, the mode shapes evaluated from Equation (5) can be used to characterize the low-frequency dynamics of the protein [35,36] and investigate its conformational changes.

In order to obtain fully quantitative information about the natural vibrational frequencies *f_n_*, we do not only need to include mass into the model but also to fix properly the values of the spring constants *γ_i,j_*. Multiple choices can be made in this regard: the traditional ANM sets *γ_i,j_* as a constant value for all connections, i.e., *γ_i,j_* = *γ* [17], while other ANM-based approaches make these values dependent on the distance between the nodes, i.e., *γ_i,j_* ∝ *r_i,j_* ^−*p*^ (*p* = 1 in [35,36], 2 in [20], in the range 0–2.8 in [18]). In this work, we use the traditional ANM convention (i.e., all springs have a unique spring constant equal to *γ*). This value can be quantified by comparing the computed fluctuations arising from the normal modes to the experimental ones, which are known as the B-factors [17,35]. B-factors constitute a fingerprint of protein flexibility and can be experimentally retrieved from crystallographic experiments. These experimental values can be compared to the ones obtained from the normal mode calculations, based on the following expression [35,53]:

(6) $$B_{i}=\frac{8}{3}\pi^{2}k_{B}T\overset{3N}{\underset{n=7}{\sum }}\frac{\delta_{n,i}{}^{2}}{\omega_{n}{}^{2}},$$

where *B_i_* represents the B-factor calculated for node *i* of the network, *k_B_* is the Boltzmann’s constant (1.38 × 10^−23^ J/K), *T* is the absolute temperature, *δ_n,i_* is the mass-weighted displacement of node *i* associated with mode *n*, and *ω_n_* is the angular frequency of mode *n*. By posing that the mean value of the calculated B-factors matches the mean value of the experimental B-factors, one is able to obtain the value of the spring constant *γ*, finally being able to obtain quantitative information about the vibrational frequencies [35,36].

### 2.2. Time-Independent Response under External Perturbations

As mentioned in the Introduction, the ANM has also been used to predict protein flexibility and conformational changes upon the application of external perturbations to the protein network [41,42,44,45,47]. This is usually done in a static fashion, meaning that dynamic effects are neglected. In this case, the protein response is obtained by solving the following matrix equation:

(7) {F}=[H]{u},

where [*H*] is the ANM Hessian matrix evaluated from Equation (2), {*F*} is a 3*N* × 1 vector of external forces and {*u*} is the 3*N* × 1 vector of nodal displacements accounting for the protein response. The choice of the force vector {*F*} depends on the specific application. For example, in [42] pairwise pulling forces are applied for each couple of residues *i* and *j*, whereas in the PRS technique a point force is usually applied at a single node [44,45] or in a localized region [46,47] with a random direction. In any case, the protein response {*u*} is computed by inverting Equation (7) as follows:

(8) $$\left\{u\right\}=[H^{-1}]\left\{F\right\},$$

where [*H*^−1^] is the pseudo-inverse Hessian matrix, which can be computed from the 3*N* – 6 eigenvalues *λ_n_* and eigenvectors {*δ_n_*} of the Hessian matrix, as follows:

(9) $$\left[H^{-1}\right]=\overset{3N}{\underset{n=7}{\sum }}\frac{1}{\lambda_{n}}\left\{\delta_{n}\right\}{\left\{\delta_{n}\right\}}^{T}.$$

The protein response {*u*} computed through Equations (7)–(9) has been often compared to the observed conformational change, and good agreement has been found in certain cases [44,45,47].

### 2.3. Time-Dependent Response under External Harmonic Perturbations

The method developed in the present work can be seen as the generalization of the techniques reported in the previous subsections, in the sense that we consider the dynamics of the system while applying external perturbations (i.e., we apply forces to the protein ANM in a dynamic fashion). In this case, we can write the full equilibrium equations as follows [52]:

(10) $$\left[M\right]\frac{d^{2}}{dt^{2}}\left\{u\left(t\right)\right\}+\left[C\right]\frac{d}{dt}\left\{u\left(t\right)\right\}+\left[H\right]\left\{u\left(t\right)\right\}=\left\{F\left(t\right)\right\},$$

where [*M*], [*C*] and [*H*] are the 3*N* × 3*N* mass, damping and Hessian matrix of the system, respectively; {*u*(*t*)} is 3*N* × 1 displacement vector representing the time-dependent protein response; and {*F*(*t*)} is the 3*N* × 1 vector of external time-dependent perturbations. Notice that when no forces act on the system and damping effects are neglected (i.e., {*F*(*t*)} = {0} and [*C*] = [0]), the problem reduces to the free-vibration analysis reported in Section 2.1. Conversely, if inertia and damping forces are neglected and the external perturbations are time-independent (i.e., [*M*] = [0], [*C*] = [0] and {*F*(*t*)} = {*F*}), the problem reduces to the calculation of the protein response under external static perturbation described in Section 2.2. If all the terms are taken into account (i.e., [*M*] ≠ [0], [*C*] ≠ [0] and d{*F*(*t*)}/d*t* ≠ {0}), Equation (10) allows to obtain the complete time-dependent protein response under external dynamic perturbations.

In this work, we apply an external pattern of forces which is random in the space-domain, but it has a harmonic content in the time-domain. Therefore, the time-dependent force vector can be written as {*F*(*t*)} = {*F*}sin(*ω_F_t*), where {*F*} is a 3*N* × 1 vector of force components randomly extracted from the uniform distribution *U*~(−1,1) × 10^−10^ N, and *ω_F_* is the angular frequency associated to the harmonic excitation, with frequency *f_F_* = *ω_F_*/2π. The protein response {*u*(*t*)} can be decoupled in the space- and time-domain through the well-known relation {*u*(*t*)} = [*Δ*]{*p*(*t*)}, where [*Δ*] is the 3*N* × 3*N* matrix containing the eigenvectors {*δ_n_*} obtained via modal analysis from Equation (5), and {*p*(*t*)} is the 3*N* × 1 vector of principal coordinates associated with each normal mode {*δ_n_*}. In this way, Equation (10) can be rewritten as [52]:

(11) $$\left[M\right]\left[\Delta \right]\frac{d^{2}}{dt^{2}}\left\{p\left(t\right)\right\}+\left[C\right]\left[\Delta \right]\frac{d}{dt}\left\{p\left(t\right)\right\}+\left[H\right]\left[\Delta \right]\left\{p\left(t\right)\right\}=\left\{F\right\}\sin\left(\omega_{F}t\right).$$

Pre-multiplying both sides of Equation (11) by [*Δ*]^T^, we observe that [*Δ*]^T^[*M*][*Δ*] = [*I*] and [*Δ*]^T^[*H*][*Δ*] = [*Ω*], where [*I*] is the 3*N* × 3*N* identity matrix and [*Ω*] is the 3*N* × 3*N* diagonal matrix containing the 3*N* natural angular frequencies *ω_n_^2^*. At this point, by assuming that the matrix product involving the damping matrix, [*Δ*]^T^[*C*][*Δ*], yields a diagonal matrix itself, the 3*N*-degree-of-freedom problem reported in Equation (11) can be fully decoupled into a set of 3*N* single-degree-of-freedom equations, as follows [52]:

(12) $$\frac{d^{2}}{dt^{2}}p_{n}\left(t\right)+2\xi_{n}\omega_{n}\frac{d}{dt}p_{n}\left(t\right)+\omega_{n}{}^{2}p_{n}\left(t\right)={\left\{\delta_{n}\right\}}^{T}\left\{F\right\}\sin\left(\omega_{F}t\right),\;\;n=1,\ldots ,3N,$$

where *ξ_n_* is the dimensionless damping coefficient associated with the *n*^th^ mode of vibration {*δ_n_*}. Since we are only interested in the internal deformation of the protein, we can focus on the 3*N* – 6 set of equations related to the non-rigid motions, i.e., *n* = 7, …, 3*N*. Considering underdamped conditions, i.e., *ξ_n_* < 1, the general solution of Equation (12) is:

(13) $$p_{n}\left(t\right)=e^{-\xi_{n}\omega_{n}t}\left[A_{n}\cos\left(\omega_{d,n}t\right)+B_{n}\sin\left(\omega_{d,n}t\right)\right]+C_{n}\sin\left(\omega_{F}t+\phi_{n}\right),$$

where the first term in the right-hand side represents the solution of the general integral, which is associated with the decaying damped response, while the latter represents the solution of the particular integral, which is related to the steady-state response due to the external harmonic excitation at frequency *ω_F_* [52], and *ω_d,n_* is the reduced frequency of mode *n*, i.e., *ω_d,n_^2^* = *ω_n_^2^*(1–*ξ_n_^2^*). The amplitude *C_n_* and phase *ϕ_n_* of the steady-state response can be written as [52]:

(14) $$C_{n}=\frac{{\left\{\delta_{n}\right\}}^{T}\left\{F\right\}}{\omega_{n}{}^{2}\sqrt{{\left(1-\beta_{n}{}^{2}\right)}^{2}+{\left(2\xi_{n}\beta_{n}\right)}^{2}}},\;\;\phi_{n}=-\arctan\left(\frac{2\xi_{n}\beta_{n}}{1-\beta_{n}{}^{2}}\right),$$

where *β_n_* is the ratio between the frequency of applied excitation *ω_F_* and the one of the *n*^th^ normal mode *ω_n_* (i.e., *β_n_* = *ω_F_*/*ω_n_*). The integration constants *A_n_* and *B_n_* depend on the initial conditions of the system. Assuming an initial resting condition (i.e., *p* (*t* = 0) = 0 and d*p*/d*t* (*t* = 0) = 0), one obtains:

(15) $$A_{n}=-C_{n}\sin\phi_{n},\;\;B_{n}=\frac{\xi_{n}\omega_{n}A_{n}-C_{n}\omega_{n}\cos\phi_{n}}{\omega_{d,n}}.$$

The numerical computation of Equations (13)–(15) allows to obtain the complete temporal evolution of the vector of principal coordinates {*p*(*t*)} and, therefore, the vector of time-dependent displacements of the protein nodes {*u*(*t*)} = [*Δ*]{*p*(*t*)}.

### 2.4. Model Parameters, Comparison between the Protein Response and the Observed Conformational Change and Principal Component Analysis (PCA)

Besides the mass, damping and stiffness features of the protein network, the time-dependent response {*u*(*t*)} depends on the applied force vector {*F*} and frequency of excitation *ω_F_*. As mentioned above, the force vector has been chosen as a random vector, whose components are picked up from a uniform distribution in the range (−1,1) × 10^−10^ N. The simulation has been run for 100 different random force patterns, in order to assess the influence of the specific force pattern on the protein response. As for the value of the frequency *f_F_*, 500 different values have been used in the range 0.001–0.5 THz, in order to investigate the influence of the excitation frequency on the response of the elastic network. The mass values have been set based on the actual amino acid atomic weights. The elastic network has been built from the protein crystal coordinates obtained from the PDB, and by adopting a cutoff value *r_c_* of 15 Å. All springs have been set the same stiffness value *γ*, which has been defined based on the quantitative comparison between the experimental and numerical B-factors, as explained in Section 2.1. Finally, since the damping characteristics of the system are not easy to understand, as a preliminary testing condition we worked in the underdamped limit, by carrying out the analysis with three different values of the dimensionless damping coefficients *ξ_n_*, i.e., *ξ_n_* = 0.001, 0.01 and 0.1. This value has also been kept equal for all modes. Discussions about the effect of damping and the reasonableness of these choices are addressed below. In conclusion, for a certain protein structure, 150,000 different simulations have been run, by considering 100 random force patterns, 500 different excitation frequencies and 3 different damping coefficients.

After the evaluation of the induced time-dependent displacement field {*u*(*t*)}, this was compared with the experimentally-observed conformational change. The conformational change can be characterized by a 3*N* × 1 vector of nodal displacements {*CC*}, which is evaluated from the two crystal protein conformations available on the PDB (usually referred to as the “open” and “closed” form of the protein) after superposition [24]. The comparison can be quantitatively assessed by calculating the overlap between the two vectors {*u*(*t*)} and {*CC*}, which is defined as [24]:

(16) $$O\left(t\right)=\frac{\left|{\left\{u\left(t\right)\right\}}^{T}\left\{CC\right\}\right|}{\sqrt{{\left\{u\left(t\right)\right\}}^{T}\left\{u\left(t\right)\right\}}\cdot \sqrt{{\left\{CC\right\}}^{T}\left\{CC\right\}}}.$$

The overlap defined in Equation (16) provides a numerical estimate of the alignment between the calculated displacements {*u*(*t*)} and the conformational change {*CC*}. If the two vectors are perfectly aligned, the overlap reaches the maximum value of 1, whereas it provides a value of 0 if the two vectors are orthogonal. In the previous literature, the overlap has been extensively used to assess the directionality correlation between the conformational change and the individual normal modes of vibrations [24,28,29]. In that case, since each normal mode of vibration has a fixed direction over time, the overlap is time-independent. Conversely, in this case, we are comparing the observed conformational change to the time-dependent response of the protein network {*u*(*t*)}, thus the overlap changes over time as the displacement field evolves in the time-domain. From the analysis of the obtained overlap values, we can infer whether the induced time-dependent protein response is in agreement with the experimentally-observed conformational change.

Principal component analysis (PCA) is also used here to investigate the generated ensemble of protein conformations. PCA is a numerical technique widely adopted for dimensionality reduction [54], and it has also been used to evaluate the apparent motions of proteins from a set of experimental crystal structures [33,55]. The input of PCA is a matrix of coordinates [*X*], with dimension *s* × 3*N*, *s* being the number of available structures and *N* the number of protein residues. In our case, *s* is equal to the number of generated protein conformations upon harmonic perturbations. From [*X*], the covariance matrix [*Σ*] can be calculated as [33]:

(17) $$\Sigma_{i,j}=〈(r_{i}-〈r_{i}〉)(r_{j}-〈r_{j}〉)〉,$$

where *r_i_* and *r_j_* represent the X-, Y- and Z-coordinates associated with conformation *i* and *j*, respectively, and < > stands for the average over all the conformations. The covariance matrix [*Σ*] is then decomposed as:

(18) $$\left[\Sigma \right]=\left[P\right]\left[\Lambda \right]{\left[P\right]}^{T},$$

where [*Λ*] is the 3*N* × 3*N* diagonal matrix of eigenvalues and [*P*] is the 3*N* × 3*N* matrix of eigenvectors. Each column of [*P*] represents a principal component (PC), ordered for descending order of its total variance, which is directly proportional to the corresponding eigenvalue included in [*Λ*]. PCA is applied here to reduce the problem dimensionality and study the protein trajectory in the PC sub-space.

## 3. Results and Discussion

**Figure 1:**
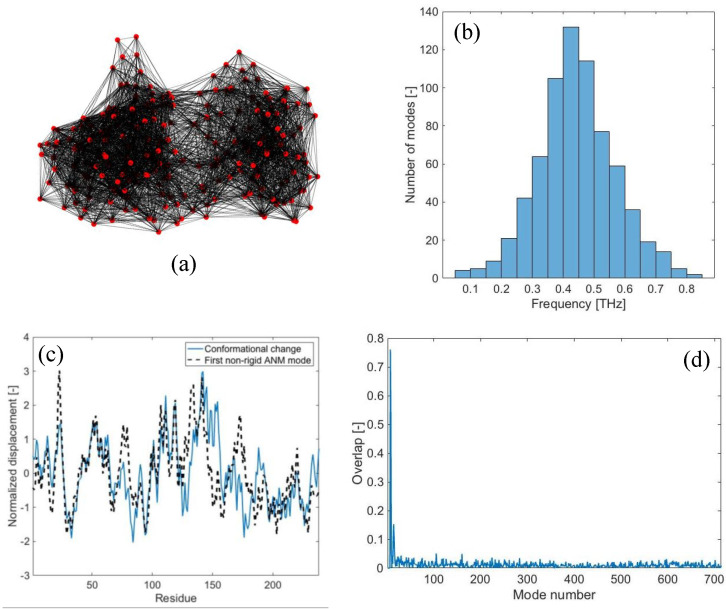
LAO-binding protein normal modes: (**a**) Elastic network model of the open conformation (PDB code: 2lao), obtained with *r_c_* = 15 Å; (**b**) distribution of vibrational frequencies obtained from free-vibration modal analysis; (**c**) normalized values of the displacements of the open-to-closed conformational change (continuous line) and displacements associated with the first non-rigid normal mode (dashed line); (**d**) overlap values obtained from the comparison of the open-to-closed conformational change to each normal mode of vibration (maximum overlap 0.76).

In this section, the results are reported for the case of LAO-binding protein, a widely studied protein, known to exhibit two different conformations (i.e., an open form (PDB code: 2lao) and a closed form upon ligand-binding (PDB code: 1lst)) [24]. In the Supplementary Material, the results for other three proteins are reported (i.e., maltodextrin-binding protein (PDB codes: 1omp, 1anf), lactoferrin (PDB codes: 1lfh, 1lfg) and triglyceride lipase (PDB codes: 3tgl, 4tgl)). The coordinates of the open form are used to build the elastic network model, with a cutoff of 15 Å (Figure 1a). Free-vibration modal analysis is run first, in order to obtain the theoretical B-factors from Equation (6) and the value of the spring constant *γ*, which is found to be equal to 0.10 N/m (~0.15 kcal/molÅ^2^). As a result, the frequency spectrum of the 3*N* – 6 (*N* = 238) non-rigid modes related to the coarse-grained elastic network is found to lie in the range 0.05–0.8 THz (Figure 1b), the lowest frequency being equal to 50.9 GHz.

Figure 1c shows the displacement field involved in the open-to-closed conformational change (continuous line). By carrying out the traditional overlap comparison between the displacement field {*CC*} and each individual normal mode {*δ_n_*}, it is found that the first non-rigid normal mode (i.e., {*δ_7_*}) is the one exhibiting the highest overlap value (0.76), as shown in Figure 1c,d. The second low-frequency mode {*δ_8_*} agrees with the conformational change with an overlap of 0.55, while all the higher-frequency modes have lower overlap scores (Figure 1d). From these results, it is clear that the low-frequency modes are strictly related to the observed conformational change, as already reported in the previous literature [24,29].

**Figure 2:**
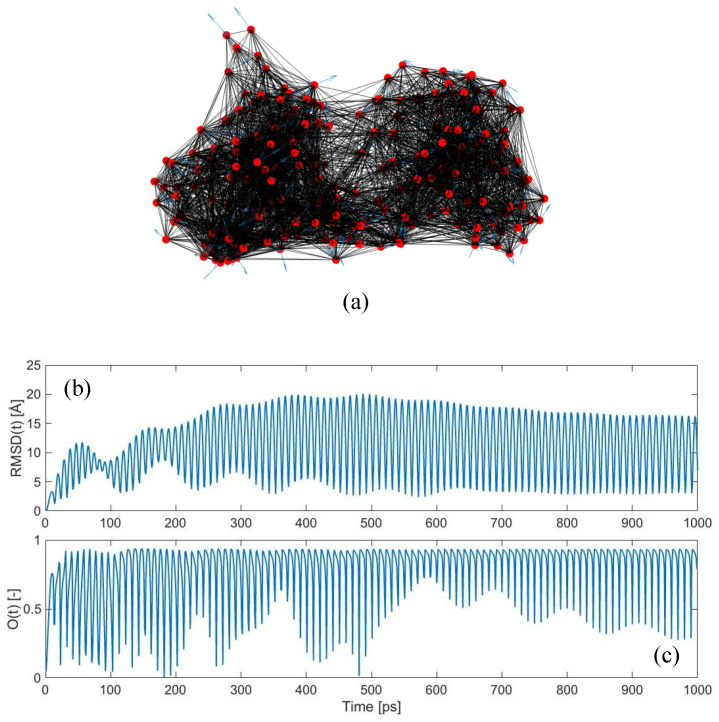
Dynamic force application on LAO-binding protein: (**a**) Elastic network model of the open conformation, with applied external forces with random directions (shown as blue arrows). Exciting frequency of the external perturbation equal to 0.05 THz and damping coefficient *ξ* equal to 0.01; (**b**) evolution of RMSD in the time-domain; (**c**) overlap values between the conformational change {*CC*} and the time-dependent displacement vector {*u*(*t*)} at each instant *t* (maximum overlap 0.94).

The results reported above are based on the traditional analysis aimed at evaluating the similarity between the individual mode shapes of the protein structure and its conformational change [24]. What happens if we look at the complete time-dependent protein response upon harmonic random perturbations, as described in Section 2.3? Figure 2 shows the time-dependent response of LAO-binding protein, subjected to the random force pattern reported in Figure 2a, with an exciting frequency of 0.05 THz and a damping coefficient *ξ* of 0.01. The response is reported in Figure 2b in terms of the global root-mean-squared-deviation (RMSD). The RMSD is a measure of the average displacements of the atoms from the initial position. It can be simply computed as:

(19) $$RMSD\left(t\right)=\sqrt{\frac{1}{N}\overset{N}{\underset{i=1}{\sum }}u_{i}{\left(t\right)}^{2}},$$

where *u_i_*(*t*) is the absolute displacement of the *i*^th^ node at instant *t*. As can be noticed from Figure 2b, the response of the protein network exhibits a transitory response at the beginning, and then enters a steady-state oscillation approximately from 400 ps onwards. Note that, with the frequency of the external oscillation of 0.05 THz, its period is equal to 20 ps. Moreover, since this frequency value is very close to the natural frequency of the first mode (*f_7_* = 0.051 THz), high amplifications in the response occur, leading to RMSD values of about 20 Å (Figure 2b). On the other hand, if we applied the same force pattern in a static way (i.e., by following the approach reported in Section 2.2), we would obtain a total RMSD of about 2.3 Å. This leads to a dynamic amplification value, evaluated as the ratio between the dynamic RMSD and static RMSD, of about 8.4. Such dynamic amplification factors might also explain why protein vibrations and responses under external forces, which are supposed to be theoretically valid only in the small-amplitude regime, might actually trigger large-scale conformational changes.

Figure 2c shows the time-dependent overlap, obtained by comparing the calculated displacement field {*u*(*t*)} with the conformational change {*CC*}, as described in Section 2.4. The results are astonishing, since values as high as 0.94 are frequently met. This unequivocally suggests that, even if the applied force pattern is completely random in the space-domain (Figure 2a), its dynamic application is able to drive the protein structure towards the known closed conformation, with remarkably high levels of agreement.

Additionally, it is interesting to observe how the overlap score is not maintained to these high values constantly, but it keeps oscillating between low and high values. This suggests that the direction of the protein motion {*u*(*t*)} from the open towards the closed conformation is not linear—see Equation (16). As a matter of fact, if this motion were linear, we would find a roughly constant value of the overlap throughout the entire simulation. The fact that this does not happen suggests that, while jiggling around its equilibrium position, the protein is sampling a variety of different conformations, among which lies the known closed form. These dynamic jumps between conformations happen in a continuous fashion and involve curvilinear pathways, as suggested here from our overlap calculations and already reported by previous authors [28,56,57,58,59,60].

**Figure 3:**
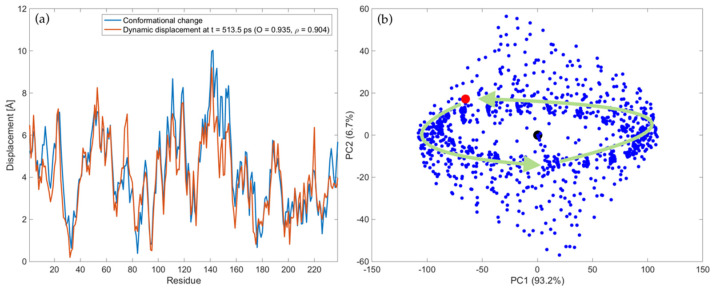
Trajectory of LAO-binding protein upon harmonic perturbations to the open form, with *f_F_* = 0.05 THz, *ξ* = 0.01 and force pattern depicted in Figure 2a: (**a**) Comparison between the displacements of the experimental conformational change (blue line) and dynamic displacements evaluated at *t* = 513.5 ps (orange line); (**b**) PC score plot of all conformations. The blue points represent the ensemble of generated conformations, whereas the black and red point represent the open (pdb: 2lao) and closed (pdb: 1lst) conformation, respectively. The green arrows are associated with the trajectory in the steady-state regime. A scale factor of 0.35 has been applied to the force pattern in order to have comparable values of absolute displacements. See Supplementary Movies S1 and S2 for more details about the protein trajectory in the coordinate and PC space, respectively.

The complete trajectory of LAO-binding protein upon the force pattern shown in Figure 2a is represented in the Supplementary Movie S1, which is available in the Supplementary Materials. In the movie the blue structure refers to the protein conformation generated at each instant *t* starting from the open form. Conversely, the red structure refers to the known closed form of the protein and it is kept fixed in all frames to help the visualization of the conformational change. As can be seen, after the motion enters in the steady-state regime, the perturbed protein structure keeps oscillating between open and closed conformations. Note that several times the known closed conformation (in red) is reached with high accuracy during the motion. The instants at which this occurs are the ones where high levels of overlap values have been obtained and reported in Figure 2c. As an example, Figure 3a shows the snapshot of the dynamic displacements evaluated at *t* = 513.5 ps, compared with the displacements of the known open-to-closed conformational change. The overlap value and correlation coefficient between the two displacement fields are 0.935 and 0.904, respectively, showing high level of agreement. Higher than that found by following only the first normal mode of vibration (compare Figure 3a with Figure 1c).

In order to describe more quantitatively the ensemble of generated conformations, PCA has been applied to the set of structures obtained during the trajectory according to Equations (17) and (18). Figure 3b reports the PC score plot of all conformations in the PC1-PC2 sub-space. Note that PC1 and PC2 account for 93.2% and 6.7% of the total variance, thus they account for 99.9% of the total variance. The black point refers to the open form (pdb: 2lao), the red point to the closed form (pdb: 1lst), while all the points associated with the generated conformations are in blue. A dynamical representation of Figure 3b can be observed in the Supplementary Movie S2, where the time-dependent evolution of the conformations in the PC1-PC2 plot is reported. From the movie and Figure 3b we see that, after a transitory, the steady-state trajectory implies a harmonic motion of the protein around the open form, mostly along the first PC (green arrows in Figure 3b). The information contained in Supplementary Movies S1 and S2 also suggests that the direction of PC1 involves mostly an opening-closing mechanism of the protein. During this harmonic oscillation, the closed conformation (red point in the PC score plot) is closely approached several times throughout the motion.

It is also interesting to observe that, even though we are applying forces at a frequency very close to the first natural mode (*f_F_* = 0.05 THz and *f_7_* = 0.051 THz), the time-dependent displacement field contains the information about the complete dynamics of the system, and not only that of the first natural mode. This can be immediately understood if one looks at the overlap values. By considering the trajectory which would be induced by the first natural mode alone, we would get a 0.74 overlap with the conformational change for the entire trajectory (Figure 1d). On the other hand, applying forces dynamically excites all modes and eventually leads to a much higher agreement with the conformational change, with *O_max_* = 0.94 (Figure 2c and Figure 3a).

Here, it is also important to notice that the methodology developed in this work does not require any a priori knowledge of the target conformation. The closed conformation is only used to assess whether the conformations generated by perturbing dynamically the reference structure overlap properly with the target. Other methods have been developed in the existing literature based on the ANM, in order to find intermediate conformations given the two end structures [47,56,57,59,61,62]. Conversely, the methodology presented here relies only on the knowledge of the reference structure and aims at evaluating its dynamic response upon external harmonic perturbations. As a result, the generated conformations do not depend on the target form, but only on the intrinsic dynamics of the reference structure and how it responses to external perturbations. Nevertheless, the conformations generated by following this approach are able to reach the other form of the protein known experimentally with high levels of agreement (see Figure 3a).

**Figure 4:**
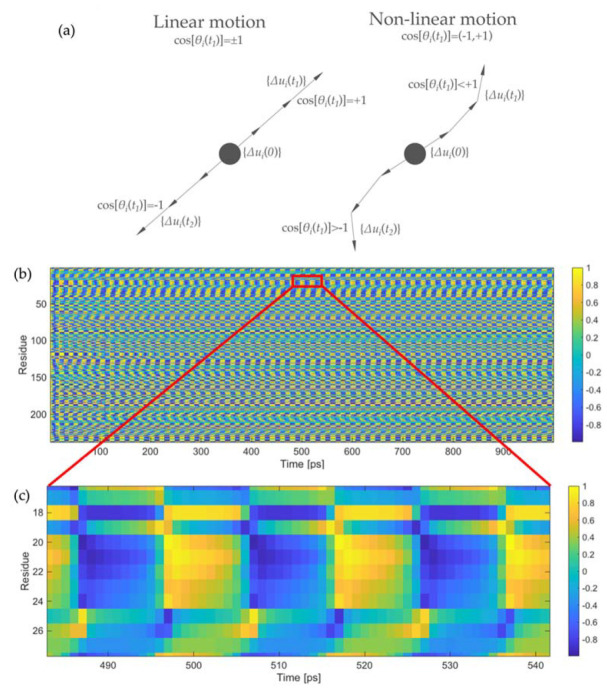
Evaluation of the motion non-linearity: (**a**) Sketch of the vectors for the numerical evaluation of motion non-linearity; (**b**) values of the normalized cosine reported in Equation (20) for the 238 residues of LAO-binding protein for the entire simulation; (**c**) enlarged portion of Figure 4b for the segment 17–27 and in the time frame between 480 and 540 ps. The value of the normalized cosines is reported in color scale, from dark blue (cosine equal to −1) to bright yellow (cosine equal to +1).

We have briefly mentioned above that the oscillating trend of the overlap values is a fingerprint of the non-linearity of the protein motion. This can also be assessed by a geometrical evaluation, as reported in Figure 4a. For each residue *i*, the coordinate difference between two subsequent conformations at time *t* and *t + Δt* provides the direction of the instantaneous motion at each time frame {*Δu_i_*(*t*)} [59]. By calculating the normalized cosine between vector {*Δu_i_*(*t*)} and the direction of motion at time *t* = 0 ps, i.e., {*Δu_i_*(0)}, as:

(20) $$\cos\left[\theta_{i}\left(t\right)\right]=\frac{{\left\{\Delta u_{i}\left(t\right)\right\}}^{T}\left\{\Delta u_{i}\left(0\right)\right\}}{\sqrt{{\left\{\Delta u_{i}\left(t\right)\right\}}^{T}\left\{\Delta u_{i}\left(t\right)\right\}}\cdot \sqrt{{\left\{\Delta u_{i}\left(0\right)\right\}}^{T}\left\{\Delta u_{i}\left(0\right)\right\}}},$$

we can geometrically evaluate the non-linearity of the trajectory. If the motion were completely linear throughout the entire simulation, the cosine would only assume values +1 and −1, the former when the protein moves in the positive direction and the latter when it comes back (Figure 4a). Conversely, non-linear motions imply cosine values different from unity, which are also supposed to change during the simulation (Figure 4a). The more frequent the change, the stronger the non-linearity of the motion. Figure 4b shows the values of the normalized cosine for all 238 residues of LAO-binding protein for the entire simulation. As can be seen, the cosine values assume all possible values in the range between −1 (dark blue) and +1 (bright yellow), suggesting that the motion is non-linear. Moreover, this variation appears to be cyclical, confirming what already observed visually from the Supplementary Movie S1, namely that the motion presents a strong harmonic nature. Figure 4c reports an enlargement of Figure 4b for residues 17–27 (the highly flexible flap of LAO-binding protein in the first domain) in the time range between 480 and 540 ps. This figure shows more clearly that each residue experiences a wide range of cosine values between −1 and +1, thus the motion is non-linear.

**Figure 5:**
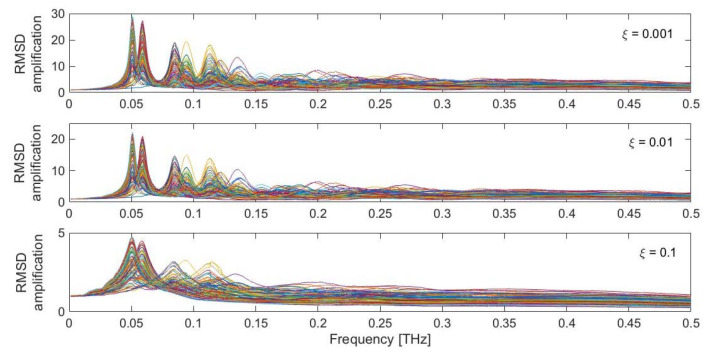
RMSD dynamic amplification for the LAO-binding protein response, as a function of damping (*ξ* = 0.001, 0.01, 0.1) and different random force patterns. Each colored curve represents one of the 100 different random force patterns.

In the analysis reported above, the protein was perturbed with a specific random force pattern (Figure 2), pulsing at a selected frequency (*f_F_* = 0.05 THz) and with a defined damping coefficient (*ξ* = 0.01). What happens if these three variables are modified? Figure 5 shows the obtained RMSD dynamic amplification, computed as the ratio between the maximum dynamic RMSD and the RMSD obtained under the application of the perturbation in a static fashion, for the whole investigated frequency range (0.001–0.5 THz) and for the three selected damping coefficients *ξ* (0.001, 0.01 and 0.1). The different colored curves are associated with each of the 100 different random force patterns applied to the protein structure.

Clear peaks are recognizable in the low-frequency range, around 0.05–0.15 THz, where the low-frequency protein modes are found to occur (Figure 1b). The intensity of the peaks is highly dependent on the adopted value of the damping coefficient. Very low values of *ξ*, such as 0.001 and 0.01, lead to amplifications of the order of 20–30. Conversely, amplification coefficients lower than 5 are found for higher damping coefficients. It is also evident that the most intense peaks are associated with the first low-frequency modes, in the region 0.05–0.06 THz. Other pronounced peaks are also found for higher modes, especially in the region between 0.08 and 0.15 THz. In the higher region of the spectrum, the dynamic amplification gets lower, eventually leading to de-amplified responses (i.e., with an RMSD amplification factor lower than 1), especially in the presence of higher damping coefficients (*ξ* = 0.1). Moreover, it can be seen that the specific force pattern has an influence on the overall system amplification. Nevertheless, highly amplified responses are always found in the low-frequency range (Figure 5). As briefly mentioned above, this dynamic amplification might be one underlying reason which enables the protein to achieve the large-scale conformational changes when it gets triggered in the low-frequency range, despite the theory behind all these calculations being strictly valid in the small-amplitude regime.

**Figure 6:**
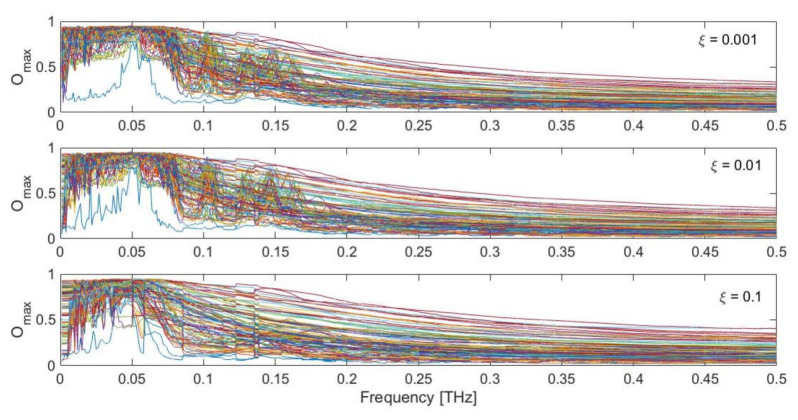
Maximum obtained overlap score for the LAO-binding protein response with respect to the observed conformational change, as a function of damping (*ξ* = 0.001, 0.01, 0.1) and different random force patterns. Each colored curve represents one of the 100 different random force patterns.

**Figure 7:**
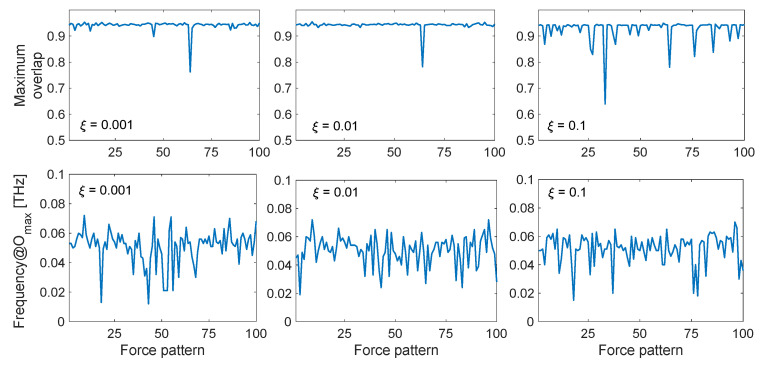
Maximum overlap score and corresponding applied frequency *f_F_* for the LAO-binding protein conformational change, as a function of damping (*ξ* = 0.001, 0.01, 0.1) and specific random force pattern. The maximum overlap values, shown in the upper panels, are defined as the maximum values obtained over all the applied frequencies in the range 0.001–0.5 THz, and the corresponding optimal frequencies are reported in the lower panels depending on each of the 100 random force patterns.

Figure 6 shows the maximum overlap values obtained during ten cycles of dynamic perturbation by comparing the calculated displacement field {*u*(*t*)} with the observed conformational change {*CC*}, as a function of the exciting frequency, damping coefficient and specific force pattern. As can be seen, remarkably high values up to 0.95 are found in the low-frequency range, especially between 0.02 and 0.08 THz. It is interesting to observe how in this low-frequency range the maximum overlap score is always very high, despite the specific force pattern. The upper panel of Figure 7 shows the maximum overlap scores obtained for each of the 100 different force patterns, for each selected damping coefficient, while the lower panel reports the exciting frequency in correspondence of which the best overlap is met. As can be seen, despite the specific random force pattern, very high values of the overlap are always obtained (up to 0.95) with applied frequencies in the range 0.02–0.08 THz, which corresponds to the low-frequency end of the spectrum (Figure 1b). From Figure 6, it can also be seen that if the protein is excited at higher frequencies, say with frequencies higher than 0.1 THz, the obtained overlap scores become lower, suggesting that the closed conformation cannot be sampled by applying harmonic excitations in this frequency range. Additionally, it can be noticed that the overlaps are higher when the damping coefficients are relatively low (*ξ* = 0.001 and 0.01).

Putting together the results obtained above, we can conclude that, by exciting the open structure with external dynamic perturbations in the low-frequency range, we can sample the closed conformation with remarkably high values of directionality correlations. In case of low damping coefficients (*ξ* = 0.001 and 0.01), one also obtains high dynamic amplification factors in that frequency range, therefore potentially allowing to reach the closed conformation even with a small amount of force involved. Finally, it also seems that the specific force pattern, which is completely random in the space-domain (Figure 2a), has not a huge influence on the results, almost always leading to high overlap scores, as long as the forces are applied with an exciting frequency in the lower part of the mode spectrum (Figure 7).

The analysis reported above focused on LAO-binding protein. However, the same analysis was carried out with other proteins (i.e., maltodextrin-binding protein, lactoferrin and triglyceride lipase; see Supplementary Material). For the maltodextrin-binding protein (PDB code of the open form: 1omp, PDB code of the closed form: 1anf, *N* = 370), one obtains a maximum overlap of 0.81 when comparing the second normal mode with the open-to-closed conformational change (Figure S1). However, overlap values as high as 0.95 can be found again when applying dynamic perturbations in the low-frequency range (Figures S3 and S4). As in the case of the LAO-binding protein, the overlaps are higher for lower damping coefficients (*ξ* = 0.001 and 0.01), which also lead to higher dynamic amplifications (Figure S2). In the case of lactoferrin (PDB code of the open form: 1lfh, PDB code of the closed form: 1lfg, *N* = 691), a maximum overlap of 0.46 is obtained between the third ANM mode and the conformational change (Figure S5). However, if the structure is perturbed dynamically in the low-frequency range, overlap values up to 0.88 can be obtained (Figures S7 and S8). From the comparison between lactoferrin and the two previous proteins, we understand that, when the individual modes have a higher agreement with the conformational change, the full dynamic response can sample the closed conformation better. Nevertheless, even when individual modes have lower similarities (*O_max_* = 0.46 for lactoferrin), the perturbation-based dynamic response allows to achieve a better agreement with the closed conformation (*O_max_* = 0.88). Finally, in the case of triglyceride lipase (PDB code of the open form: 3tgl, PDB code of the open form: 4tgl, *N* = 265), one obtains a really low value of the overlap when comparing individual ANM modes to the conformational change (*O_max_* = 0.27 for the fourteenth mode, Figure S9). As a result, the maximum overlap found by applying dynamic perturbation to the protein ANM is only 0.44 (Figures S11 and S12), showing that, in this case, the closed conformation cannot be sampled with high accuracy by the proposed method. This shows that the method proposed here always leads to higher overlaps than those obtained through the classic individual mode comparison. However, the method works better when the low-frequency modes have already a relevant similarity with the conformational change. As Tama and Sanejouand showed in their seminal work [24], this is a direct consequence of the degree of collectivity of the conformational change. Collective transitions are usually better captured by the low-frequency modes, whereas localized conformational changes usually are not. For the four proteins investigated here, the collectivity degree of their conformational transitions are 0.68 (LAO-binding protein), 0.67 (maltodextrin-binding protein), 0.48 (lactoferrin) and 0.07 (triglyceride lipase). As a result, the LAO-binding protein and the maltodextrin-binding protein reach very high values of the overlap from the full dynamic response (0.95), lactoferrin reaches a high value (0.88), while triglyceride lipase reaches a quite low value (0.44). This leads us to conclude that, with the proposed methodology, starting from the open conformation of the protein and without any a priori knowledge of the closed form, we are able to capture the closed conformations accurately as long as the conformational transition is quite collective in nature.

In all previous examples, we have investigated the conformational change from the open to the closed conformation. Figures S13 and S14 show the results of the analysis for LAO-binding protein, this time considering the closed conformation (pdb: 1lst) as reference and the open conformation (pdb: 2lao) as target. In agreement with what said above for the open-to-closed conformational transitions, the time-dependent force application generally allows to reach higher overlap values than those obtained by comparison with individual modes. As a matter of fact, when only looking at individual modes, the third ANM mode is the one showing the highest overlap, with *O_max_* = 0.56 (Figure S13). Conversely, applying dynamic forces can enhance this maximum overlap, reaching values of *O_max_* = 0.75 (Figure S14). Again, this suggests that the target conformation can generally be captured better by considering the full dynamic response of the protein, rather than looking at the trajectory generated with individual modes. However, despite the improvement, this value is lower than the one obtained when looking at the open-to-closed conformational change (compare Figure S14 with Figure 6). This suggests that, as already noticed by Tama and Sanejouand [24] and subsequent researchers, the closed-to-open transition is generally more difficult to generate than the open-to-closed one when working with ENMs.

From the lower panels of Figure 7 and Figures S4, S8 and S12, one can also observe that there is not a unique value of the exciting frequency leading to the maximum overlap score. In fact, there exists a range of low-frequency values, where each specific force pattern is able to sample the target conformation with the highest directionality correlation. This suggests that, although we are often in the range of the first fundamental frequency (i.e., the frequency associated to the first non-rigid mode shape), the optimal excitation frequency might be slightly different than the fundamental frequency, mostly due to the not negligible involvement of higher-order modes in the definition of the complete protein dynamic response. Moreover, the exact values of these frequencies must be treated carefully, as they are strongly dependent on the model parameters, such as the cutoff and the definition of spring network [35,36]. The absolute values of these frequencies strongly depend on the value of the adopted spring constant *γ*, which in turn is defined upon comparison between the numerical and experimental B-factors (Section 2.1). In doing such direct comparison, we are implicitly assuming that the experimental B-factors are dominated by the protein fluctuations, i.e., they only depend on the internal protein dynamics. Unfortunately, this is not always the case, since studies have shown that B-factors might also include other contributions coming from rigid motions, crystal disorder, refinement effects, etc. [63,64,65,66]. Therefore, for all the above mentioned reasons, when using ENMs for the understating of protein motions and their corresponding frequencies of vibration, we can only have some insights on the expected frequency range, and not on the individual frequency values.

Additional considerations need to be done regarding the damping coefficients adopted in the present analysis. In this work, values of *ξ* = 0.001, 0.01 and 0.1 have been used, meaning that the problem is treated in the underdamped regime (*ξ* < 1). Moreover, the value of *ξ* has been kept constant for all the vibrational modes (i.e., *ξ_n_* = *ξ*). However, one should be careful about such choices. As a matter of fact, in the framework of NMA and ENMs, the dynamic response of proteins is studied with no damping at all (i.e., *ξ* = 0). Conversely, few studies using Langevin network models (LNMs) have shown that the dynamics of macromolecules and proteins might be strongly overdamped (i.e., *ξ* >> 1), at least for the lowest-frequency modes [67,68]. We might also reasonably expect that the damping characteristics change for the different modes of vibration, the lowest-frequency ones being the more damped, the highest-frequency ones the less damped [68]. By using the LNM, Miller et al. [68] showed that at normal water viscosities most of the protein modes should be overdamped. Nevertheless, recent studies based on optical Kerr-effect (OKE) spectroscopy revealed the existence of underdamped global protein vibrations in the THz frequency range [69]. Therefore, it is evident that there is still little consensus nowadays about the damping nature of these functional protein vibrations. Previous numerical results showing that undamped vibrational modes correlate well with protein conformational changes [24,28,29], as well as the outcomes of our calculations based on the underdamped assumption, seem to suggest that these functional conformational transitions can indeed be retrieved by working in the underdamped limit. Yet, extensive research efforts still need to be carried out in the future to address this open issue.

Remarks need to be given also regarding the physical meaning associated with the adopted perturbation scheme (i.e., harmonic excitations with a random direction in the space-domain but with a well-defined frequency content in the time-domain). In the previous literature, different force application patterns have been applied in a static fashion to probe protein flexibility [41,42] and protein conformational changes [44,45,47]. The approach proposed here (i.e., applying random forces to the protein ANM in a dynamic fashion) is supposed to simulate the external perturbations to the protein structure mainly due to Brownian motions of the surrounding particles [70]. These collisions can be numerically simulated as random forces both in the space- and time-domain [68]. However, we know that every time-dependent variable can be decomposed according to their frequency component (e.g., via the Fourier transform (FT) or other signal transformation techniques). In this way, a random excitation in the time-domain can always be represented as a sum of harmonic excitations at specific frequencies, weighted by their FT amplitude. Based on these considerations, the application of harmonic forces with random directions can be seen as the attempt to investigate the response of the protein structure under the different frequency-based components of the complex time-dependent excitations due to the particle collisions. From the results of the present analysis, we obtained that the low-frequency components of these excitations are able to drive the protein conformational change. Conversely, high-frequency components are not particularly relevant for the conformational transition (see Figure 6).

## 4. Conclusions

In this paper, the ANM was used for the first time in order to investigate the complete dynamic response of protein structures under external dynamic perturbations. In particular, by considering the mass, viscous damping and elastic stiffness features of the protein ENM, the equations of motions were numerically solved in order to retrieve the protein response under the effect of harmonic forces applied to the protein structure. From the results, it was observed that the application of dynamic forces to the open protein conformation in the low-frequency range is able to drive the protein towards the closed form with a high directionality alignment (overlap of 0.95). Such correlation is even higher than that usually obtained when comparing individual modes to the conformational change. This is mostly due to the fact that, when the external perturbations are applied harmonically at selected frequencies, the full dynamics of the system depends on the combination of the most involved modes, and not only on a single mode.

By analyzing the time-dependent overlap values, it was possible to recognize how the closed form of the protein is not reached through a straight line pathway, since the overlap score usually oscillates between low and high values. This suggests that the protein samples the target conformation among a variety of other conformations, and it does so by following curvilinear pathways, as already suggested in the existing literature [58,59,60]. This was also been confirmed by geometrical calculations shown in this paper, where we looked at the evolution of displacement vectors throughout the motion. The trajectory of the protein upon harmonic perturbation was also shown and PCA was used to reduce the problem dimensionality and investigate the ensemble of generated conformations in the sub-space of the PCs. Furthermore, taking into account the dynamic response of the protein network under these external forces, high dynamic amplification values are often found, especially when the perturbation is applied in the low-frequency range and for low values of the damping ratio (resonance effect). These high dynamic amplifications might explain why a combination of the normal modes of vibration, which theoretically are only valid in the small-amplitude regime, allows the target form of the protein to be reached even in the case of large-scale conformational transitions. Moreover, the dynamic nature (frequency) of the excitation seems to be the central parameter driving the conformational transition, the specific force pattern having a smaller influence on the capability of the protein to sample its target conformation.

The well-known case of the LAO-binding protein was addressed in the main text of this paper. The results related to the maltodextrin-binding protein, lactoferrin and triglyceride lipase were briefly discussed and are available in the Supplementary Material. From all the cases, we observed that external harmonic excitations in the low-frequency range always lead to higher overlaps with the observed conformational change, rather than those obtained by comparing individual modes. These overlap values are generally higher the more collective the conformational transition is. Moreover, as expected, we observed that the open-to-closed conformational change is easier to be captured than the closed-to-open one. Future research efforts will be dedicated to investigating the effect of the ENM parameters, such as the cutoff, spring constant distribution, the damping ratio, as well as the nature of protein conformational change on the obtained outcomes. Notwithstanding, the preliminary results shown here seem to suggest that the low-frequency components of the random external perturbations to the protein structure, due to the surrounding environment, might play a key role in driving the biologically-relevant conformational transition.

## Data Availability

Data are available upon request to the corresponding author.

## Supplementary Materials

The following are available online at https://www.mdpi.com/article/10.3390/ijms221910501/s1, Figures S1–S4: results from the harmonic perturbation on maltodextrin-binding protein and comparison with the open-to-closed conformational change, Figures S5–S8: results from the harmonic perturbation on lactoferrin and comparison with the open-to-closed conformational change, Figures S9–S12: results from the harmonic perturbation on triglyceride lipase and comparison with the open-to-closed conformational change, Figures S13–S14: results from the harmonic perturbation on LAO-binding protein and comparison with the closed-to-open conformational change. Supplementary Movie S1: trajectory of LAO-binding in the space-domain upon harmonic perturbations, Supplementary Movie S2: trajectory of LAO-binding protein in the sub-space of PCS upon harmonic perturbations.
