# Supplementary material for: Low-Frequency Harmonic Perturbations Drive Protein Conformational Changes
Source: Int J Mol Sci. 2021 Sep 28;22(19):10501. doi: 10.3390/ijms221910501 (PMC8508695; doi:10.3390/ijms221910501)
Supplement: Supplementary file 1 [file ijms-22-10501-s001.zip › Supplementary Material/Supplementary_material_rev.docx]

Supplementary Material

Low-frequency harmonic perturbations on elastic network models drive protein conformational changes

Domenico Scaramozzino ^1,^*, Gianfranco Piana ^1,2^, Giuseppe Lacidogna ^1^ and Alberto Carpinteri ^1,3^

^1^ Department of Structural, Geotechnical and Building Engineering, Politecnico di Torino, Corso Duca degli Abruzzi 24, 10129 Torino, Italy; domenico.scaramozzino@polito.it (D.S.), gianfranco.piana@polito.it (G.P.), giuseppe.lacidogna@polito.it (G.L.), alberto.carpinteri@polito.it (A.C.)

^2^ Department of Bridge Engineering, Tongji University, 1239 Siping Road, Shanghai, China

^3^ Department of Civil and Environmental Engineering, Shantou University, Shantou, China

* Correspondence: domenico.scaramozzino@polito.it; Tel.: +39 011 0904854 (D.S.)

| **Citation:** Scaramozzino, D.; Piana, G.; Lacidogna, G.; Carpinteri, A. Low-frequency harmonic perturbations on elastic network models drive protein conformational changes. *Int. J. Mol. Sci.* **2021**, *22*, x. https://doi.org/10.3390/xxxxx  Academic Editor: Firstname Lastname  Received: date  Accepted: date  Published: date  **Publisher’s Note:** MDPI stays neutral with regard to jurisdictional claims in published maps and institutional affiliations.  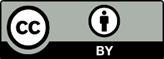  **Copyright:** © 2021 by the authors. Submitted for possible open access publication under the terms and conditions of the Creative Commons Attribution (CC BY) license (https://creativecommons.org/licenses/by/4.0/). |
| --- |

Supplementary Material

S1. Maltodextrin-binding protein (open conformation: 1omp, closed conformation: 1anf)

**Figure S1.** Maltodextrin-binding protein normal modes: **(a)** elastic network model of the open conformation (PDB code: 1omp), obtained with *r_c_* = 15 Å; **(b)** distribution of vibrational frequencies obtained from free-vibration modal analysis; **(c)** normalized values of the displacements of the open-to-closed conformational change (continuous line) and displacements associated with the second non-rigid normal mode (dashed line); **(d)** overlap values obtained from the comparison of the open-to-closed conformational change to each normal mode of vibration (maximum overlap 0.81).

**
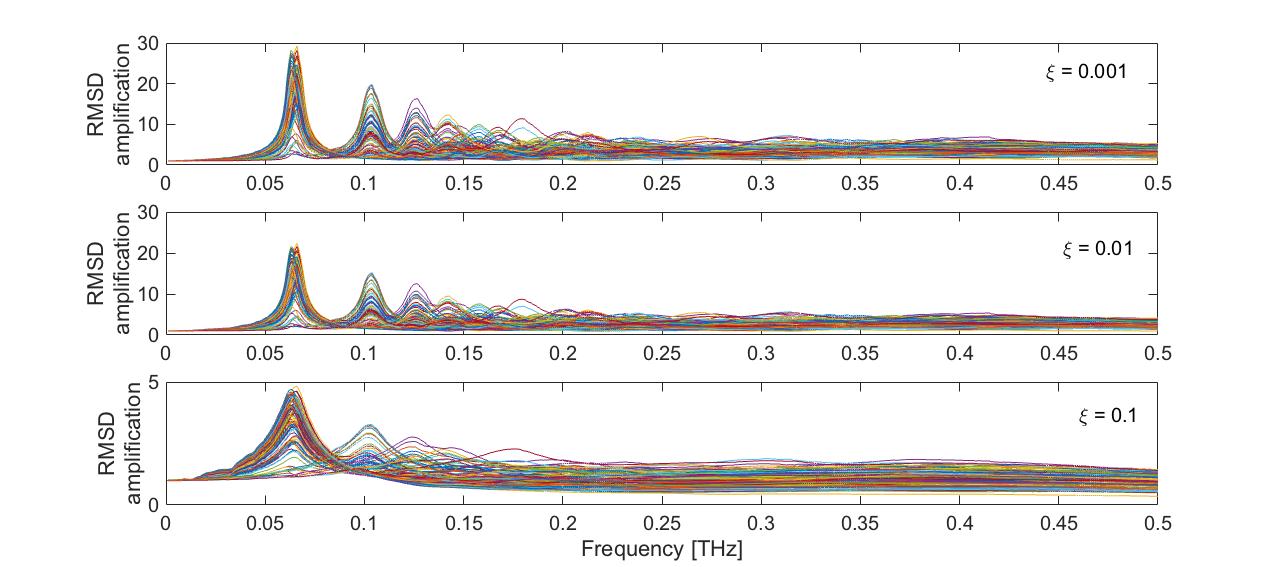
**

**Figure S2.** RMSD dynamic amplification for the maltodextrin-binding protein response, as a function of damping (*ξ* = 0.001, 0.01, 0.1) and different random force patterns. Each colored curve represents one of the 100 different random force patterns.

**
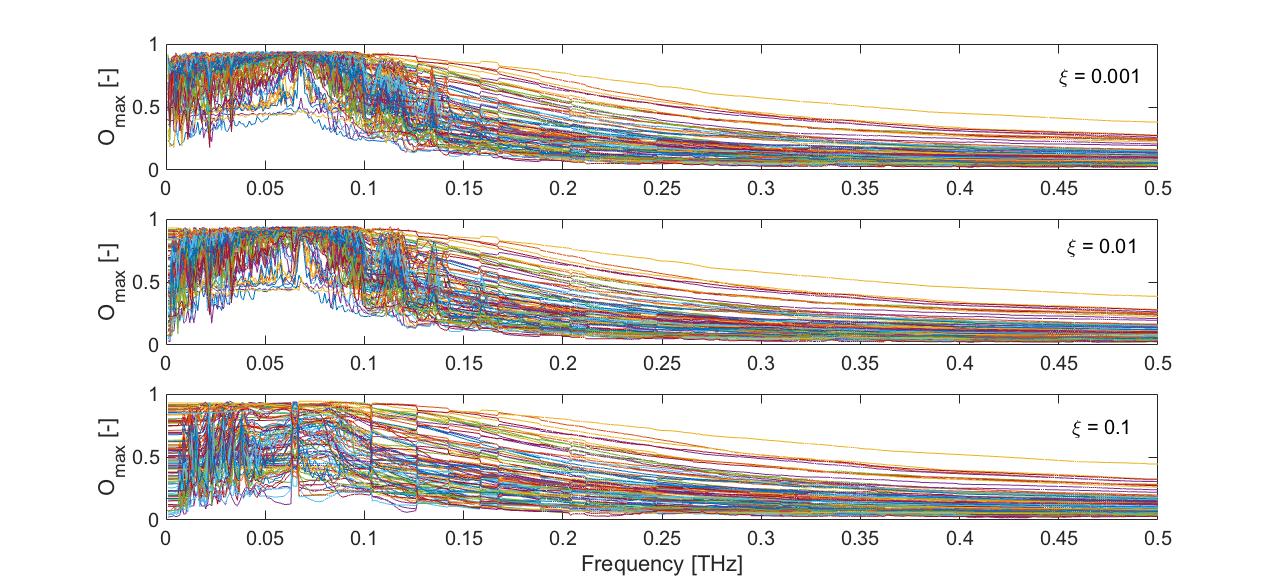
**

**Figure S3.** Maximum obtained overlap score for the maltodextrin-binding protein response with respect to the observed conformational change, as a function of damping (*ξ* = 0.001, 0.01, 0.1) and different random force patterns. Each colored curve represents one of the 100 different random force patterns.

**
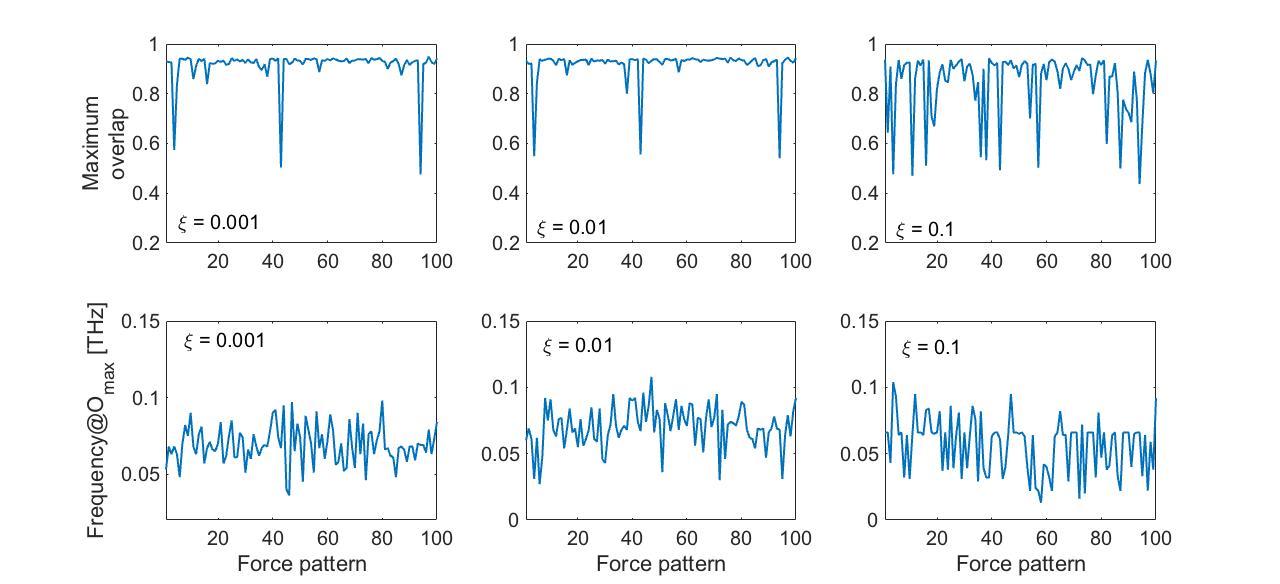
**

**Figure S4.** Maximum overlap score and corresponding applied frequency *f_F_* for the maltodextrin-binding protein conformational change, as a function of damping (*ξ* = 0.001, 0.01, 0.1) and specific random force pattern. The maximum overlap values, shown in the upper panels, are defined as the maximum values obtained over all the applied frequencies in the range 0.001 – 0.5 THz, and the corresponding optimal frequencies are reported in the lower panels depending on each of the 100 random force patterns.

S2. Lactoferrin (open conformation: 1lfh, closed conformation: 1lfg)

**Figure S5.** Lactoferrin normal modes: **(a)** elastic network model of the open conformation (PDB code: 1lfh), obtained with *r_c_* = 15 Å; **(b)** distribution of vibrational frequencies obtained from free-vibration modal analysis; **(c)** normalized values of the displacements of the open-to-closed conformational change (continuous line) and displacements associated with the third non-rigid normal mode (dashed line); **(d)** overlap values obtained from the comparison of the open-to-closed conformational change to each normal mode of vibration (maximum overlap 0.46).

**
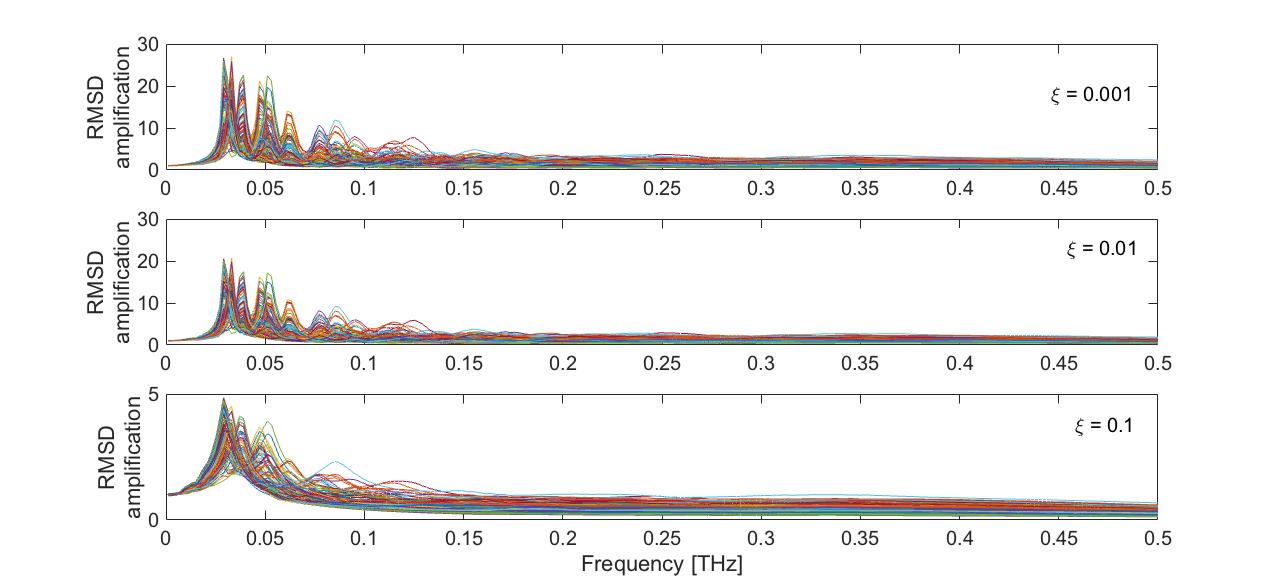
**

**Figure S6.** RMSD dynamic amplification for the lactoferrin response, as a function of damping (*ξ* = 0.001, 0.01, 0.1) and different random force patterns. Each colored curve represents one of the 100 different random force patterns.

**
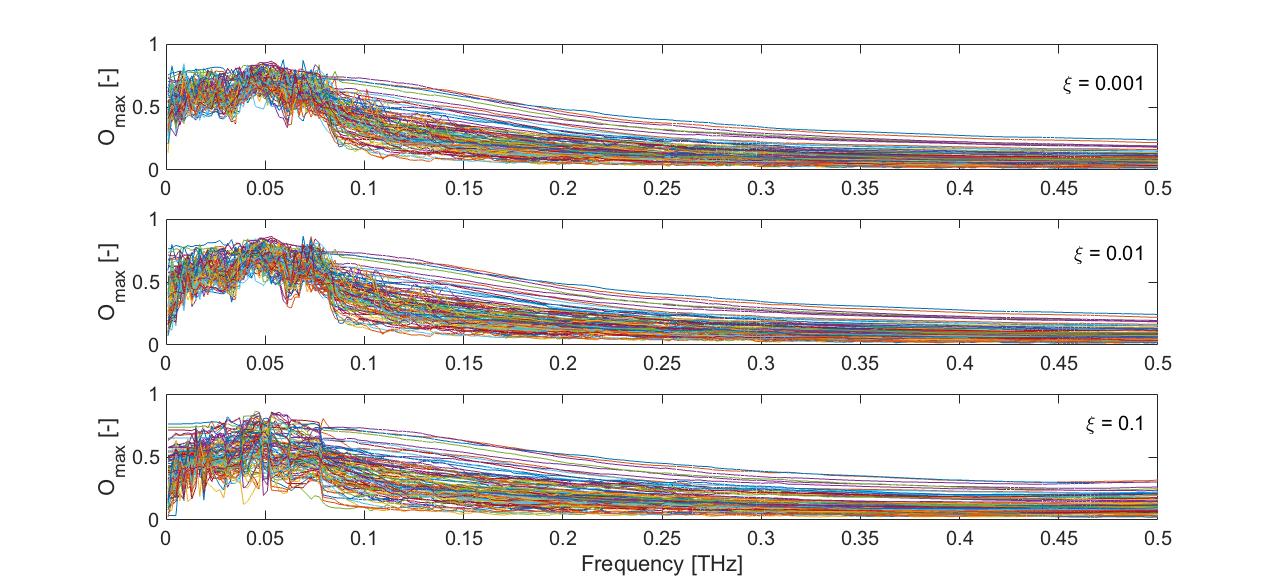
**

**Figure S7.** Maximum obtained overlap score for the lactoferrin response with respect to the observed conformational change, as a function of damping (*ξ* = 0.001, 0.01, 0.1) and different random force patterns. Each colored curve represents one of the 100 different random force patterns.


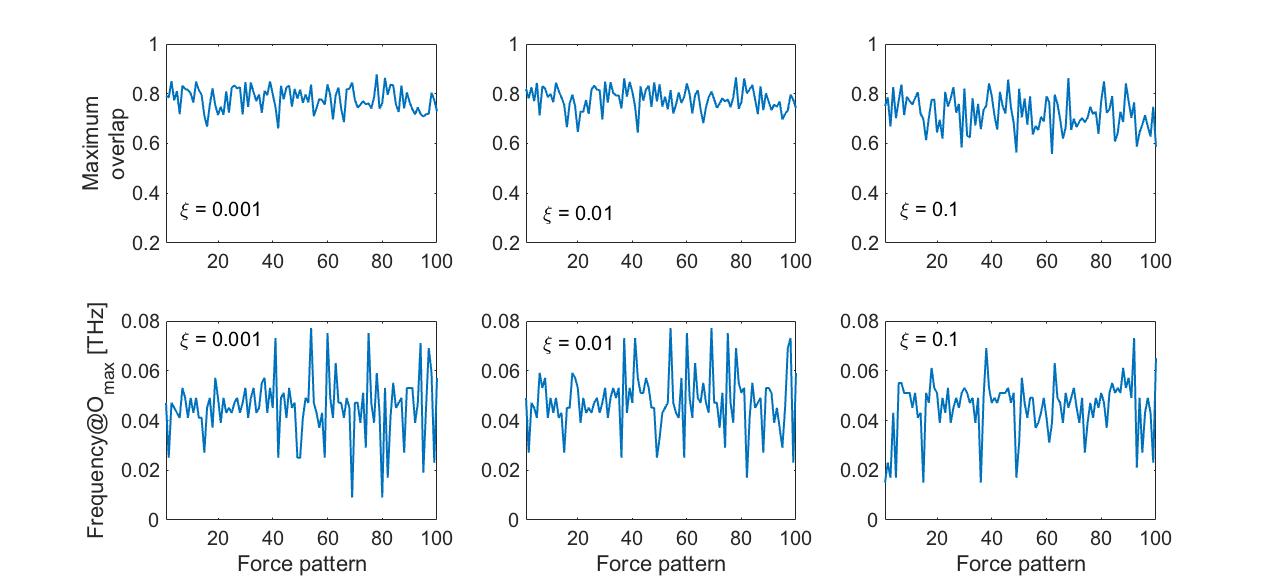


**Figure S8.** Maximum overlap score and corresponding applied frequency *f_F_* for the lactoferrin conformational change, as a function of damping (*ξ* = 0.001, 0.01, 0.1) and specific random force pattern. The maximum overlap values, shown in the upper panels, are defined as the maximum values obtained over all the applied frequencies in the range 0.001 – 0.5 THz, and the corresponding optimal frequencies are reported in the lower panels depending on each of the 100 random force patterns.

S3. Triglyceride lipase (open conformation: 3tgl, closed conformation: 4tgl)

**Figure S9.** Triglyceride lipase normal modes: **(a)** elastic network model of the open conformation (PDB code: 3tgl), obtained with *r_c_* = 15 Å; **(b)** distribution of vibrational frequencies obtained from free-vibration modal analysis; **(c)** normalized values of the displacements of the open-to-closed conformational change (continuous line) and displacements associated with the fourteenth non-rigid normal mode (dashed line); **(d)** overlap values obtained from the comparison of the open-to-closed conformational change to each normal mode of vibration (maximum overlap 0.27).

**
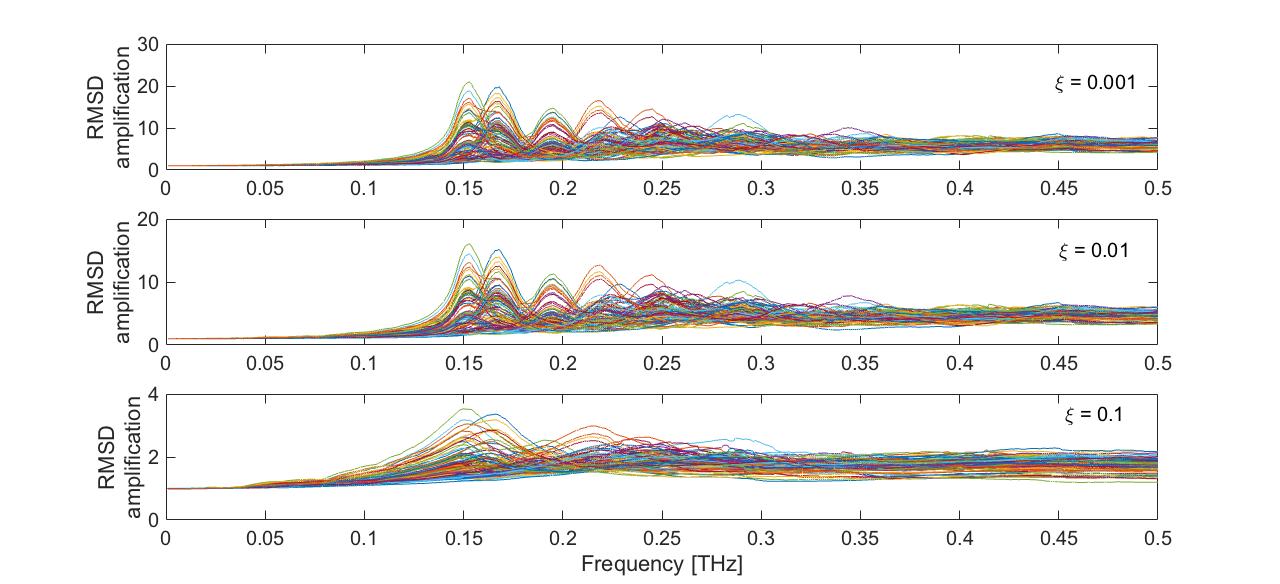
**

**Figure S10.** RMSD dynamic amplification for the triglyceride lipase response, as a function of damping (*ξ* = 0.001, 0.01, 0.1) and different random force patterns. Each colored curve represents one of the 100 different random force patterns.

**
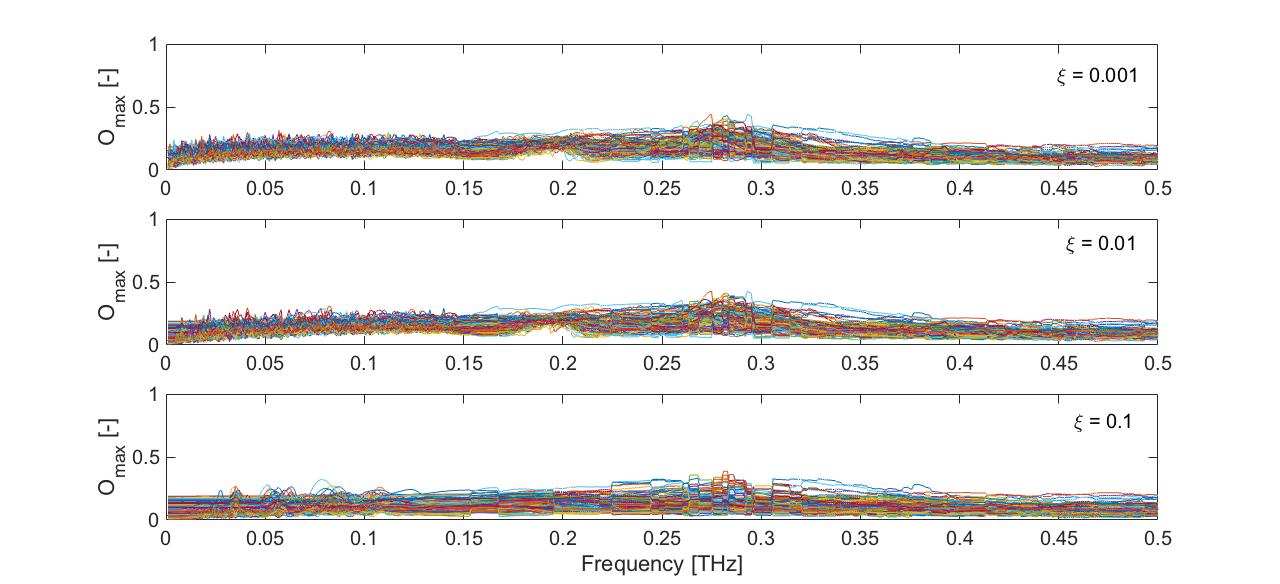
**

**Figure S11.** Maximum obtained overlap score for the triglyceride lipase response with respect to the observed conformational change, as a function of damping (*ξ* = 0.001, 0.01, 0.1) and different random force patterns. Each colored curve represents one of the 100 different random force patterns.


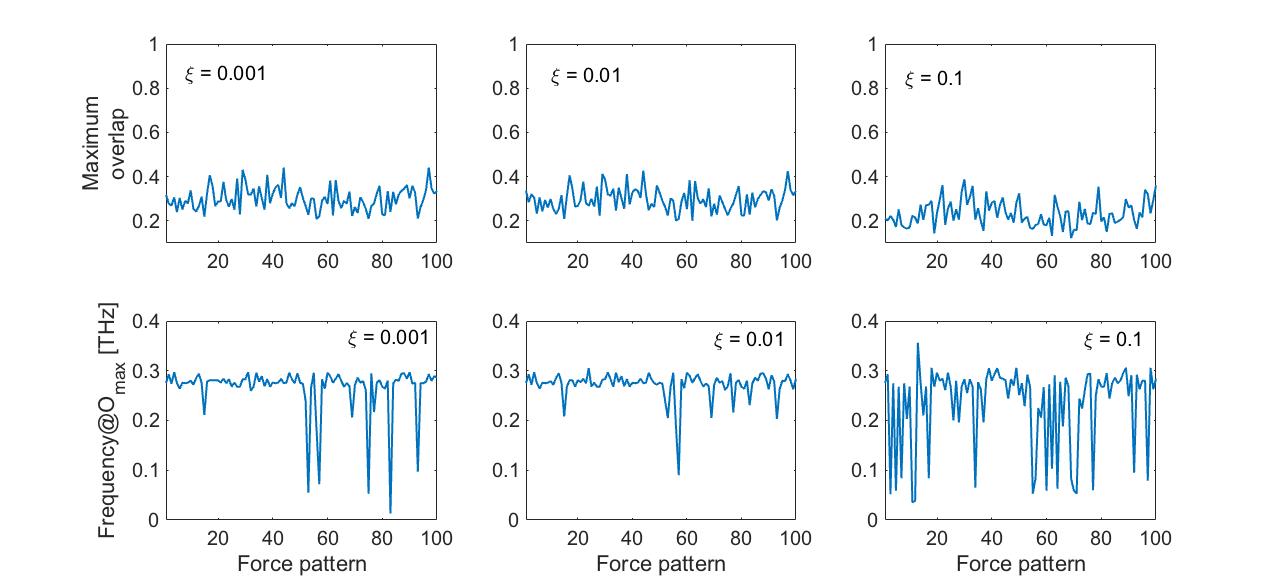


**Figure S12.** Maximum overlap score and corresponding applied frequency *f_F_* for the triglyceride lipase conformational change, as a function of damping (*ξ* = 0.001, 0.01, 0.1) and specific random force pattern. The maximum overlap values, shown in the upper panels, are defined as the maximum values obtained over all the applied frequencies in the range 0.001 – 0.5 THz, and the corresponding optimal frequencies are reported in the lower panels depending on each of the 100 random force patterns.

S4. LAO-binding protein (closed conformation: 1lst, open conformation: 2lao)

(b)

(a)

**Figure S13.** LAO-binding protein normal modes calculated on the ANM of the closed conformation (PDB code: 1lst), obtained with *r_c_* = 15 Å; **(a)** normalized values of the displacements of the closed-to-open conformational change (continuous line) and displacements associated with the third non-rigid normal mode (dashed line); **(b)** overlap values obtained from the comparison of the open-to-closed conformational change to each normal mode of vibration (maximum overlap 0.56).

**
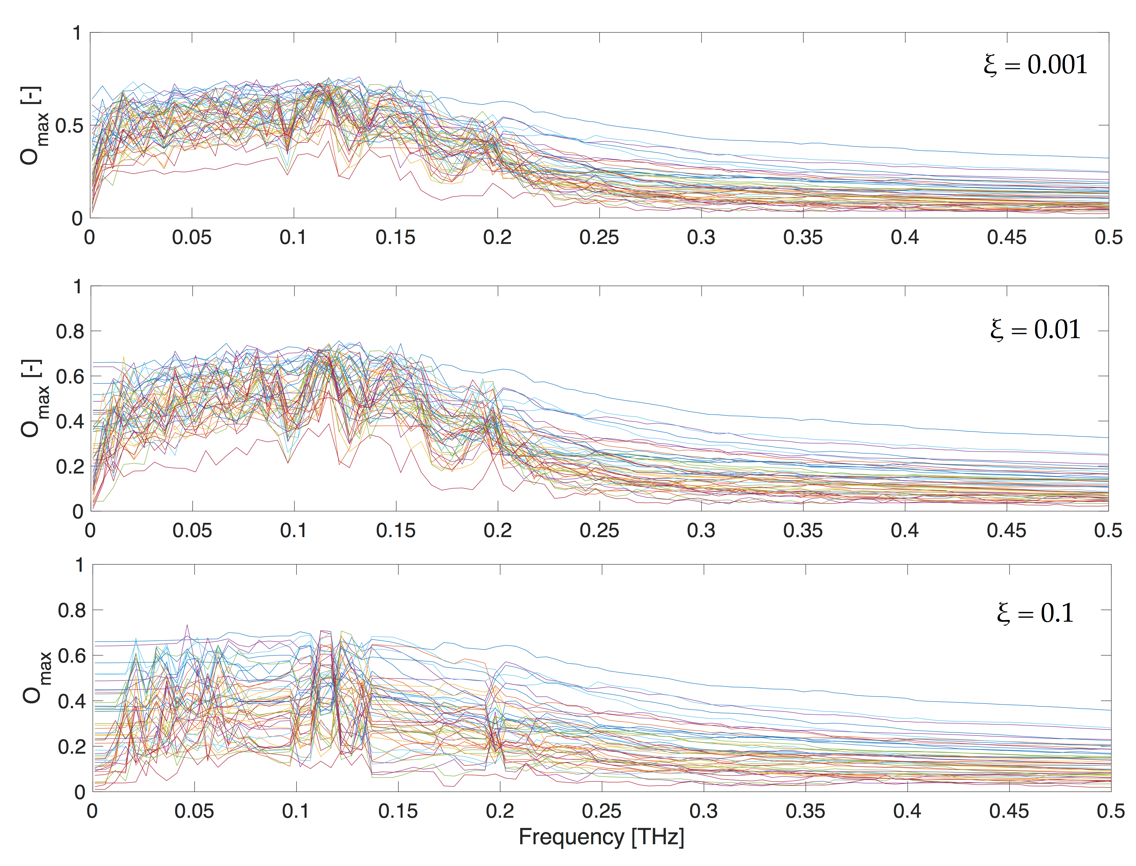
**

**Figure S14.** Maximum obtained overlap score for the LAO-binding protein response with respect to the observed closed-to-open conformational change, as a function of damping (*ξ* = 0.001, 0.01, 0.1) and different random force patterns. Each colored curve represents one of the 50 different random force patterns.
